# A Systematic Mutational Analysis of a Histone H3 Residue in Budding Yeast Provides Insights into Chromatin Dynamics

**DOI:** 10.1534/g3.115.017376

**Published:** 2015-02-23

**Authors:** Paige Johnson, Virginia Mitchell, Kelsi McClure, Martha Kellems, Sarah Marshall, Mary K. Allison, Harrison Lindley, Hoai-Trang T. Nguyen, Jessalyn E. Tackett, Andrea A. Duina

**Affiliations:** Biology Department, Hendrix College, Conway, Arkansas 72032

**Keywords:** Spt16, chromatin, histone H3, intragenic transcription initiation, transcription elongation

## Abstract

In previous work using the *Saccharomyces cerevisiae* model system, a mutant version of histone H3—H3-L61W—was found to confer a variety of abnormal growth phenotypes and defects in specific aspects of the transcription process, including a pronounced alteration in the distribution pattern of the transcription elongation factor Spt16 across transcribed genes and promotion of cryptic transcription initiation within the *FLO8* gene. To gain insights into the contribution of the H3-L61 residue to chromatin function, we have generated yeast strains expressing versions of histone H3 harboring all possible natural amino acid substitutions at position 61 (H3-L61X mutants) and tested them in a series of assays. We found that whereas 16 of the 19 H3-L61X mutants support viability when expressed as the sole source of histone H3 in cells, all 19 confer abnormal phenotypes ranging from very mild to severe, a finding that might in part explain the high degree of conservation of the H3-L61 residue among eukaryotes. An examination of the strength of the defects conferred by each H3-L61X mutant and the nature of the corresponding substituted residue provides insights into structural features of the nucleosome required for proper Spt16−gene interactions and for prevention of cryptic transcription initiation events. Finally, we provide evidence that the defects imparted by H3-L61X mutants on Spt16−gene interactions and on repression of intragenic transcription initiation are mechanistically related to each other.

Within the eukaryotic cell nucleus, DNA is condensed through the formation of chromatin, a protein−DNA complex with the nucleosome as its fundamental unit. Nucleosomes are composed of ∼147 base-pairs of DNA wrapped around the histone octamer, itself composed of a histone H3-H4 tetramer and two H2A-H2B dimers (Luger *et al.* 1997). In addition to directing DNA condensation, nucleosomes also are critically important for the regulation of processes that use DNA as template, including gene transcription. A variety of factors have been identified that can regulate specific aspects of transcription by affecting the dynamics of nucleosomes. These include enzymes that modify histone residues through the addition or removal of specific chemical groups, chromatin remodeling complexes that can alter DNA−histone interactions or the stoichiometry of canonical histones and histone variants within individual nucleosomes in an ATP-dependent manner, and histone chaperones that facilitate the assembly and disassembly of nucleosomes through mechanisms that do not rely on ATP hydrolysis (Campos and Reinberg 2009; Rando and Winston 2012).

FACT (FAcilitates Chromatin Transcription/Transactions) is a highly conserved histone chaperone complex with roles in several chromatin-based processes. In the budding yeast *Saccharomyces cerevisiae*, FACT is a heterodimer composed of the proteins Spt16 and Pob3 that can physically interact with nucleosomes with the assistance of the HMG protein Nhp6 (Brewster *et al.* 1998; Formosa *et al.* 2001; Ruone *et al.* 2003). FACT’s ability to interact with nucleosomes and alter their structure is used by cells to control chromatin environment dynamics during both the initiation and elongation phases of transcription (Reinberg and Sims 2006; Duina 2011; Winkler and Luger 2011; Formosa 2012). Arguably, the most extensively studied and better-understood aspect of FACT biology relates to its function during transcription elongation. Work by many groups during the course of the last few decades have demonstrated that FACT plays a dual role during elongation, altering nucleosome conformation ahead of transcribing RNA polymerase II (Pol II) to facilitate Pol II progression across genes and assisting in the reassembly of nucleosomes after Pol II passage (Reinberg and Sims 2006; Duina 2011; Winkler and Luger 2011; Formosa 2012). 

Several studies have provided insights into the mechanism of action of FACT in these processes. Some of these studies have culminated in a model whereby FACT−nucleosome interactions result in formation of so-called reorganized nucleosomes, in which FACT and all nucleosomal components are tethered together but whose histone subunits are more prone to dissociation during engagement with transcribing Pol II complexes (Formosa 2008; Xin *et al.* 2009). Other studies have supported a mechanism that involves specific interactions between FACT and H2A-H2B dimers as central drivers for alterations in DNA-histone interactions conducive to transcription elongation (Winkler *et al.* 2011; Hondele *et al.* 2013; Hsieh *et al.* 2013). FACT is thought to contribute to the maintenance of the epigenetic code across transcribed genes by reassembling nucleosomes using the same histone H3 and H4 subunits that were present on chromatin ahead of Pol II passage (Jamai *et al.* 2009).

A comprehensive model for the mode of action of FACT at transcribed genes requires an understanding of the physical and functional interplay between FACT and histones *in vivo*. Insights into these relationships have come in part from genetic studies in *S. cerevisiae* in which the effects of specific histone mutants on FACT function have been assessed. For example, recent work from the Formosa laboratory has shown that specific mutations predicted to disrupt the H2A-H2B dimer:H3-H4 tetramer interface suppress defects in FACT activity, a result consistent with the notion that FACT facilitates dimer-tetramer dissociation (Mccullough *et al.* 2011). In another recent study, analyses of yeast cells expressing a histone H2B mutant lacking the HBR domain—a conserved region within the N-terminus of the protein––revealed a requirement for this domain in promoting FACT-mediated H2A-H2B eviction from nucleosomes (Zheng *et al.* 2014).

We have previously identified specific histone mutants—H3-L61W, H4-R36A, and H4-K31E—that markedly impair the ability of the FACT subunit Spt16 to properly associate with chromatin (Duina *et al.* 2007; Nguyen *et al.* 2013). These mutants cause decreased levels of Spt16 occupancy at the 5′ regions of transcribed genes and increased levels at the 3′ regions of the same genes, resulting in an overall shift in Spt16 distribution toward the 3′ ends of genes. Because H3-L61, H4-R36 and H4-K31 are located near each other on the side of nucleosomes, we have proposed that these residues define a nucleosomal region important for proper Spt16−gene interactions, possibly by participating in physical interactions with Spt16 during transcription (Nguyen *et al.* 2013). In this study, we have carried out a systematic mutational analysis of the H3-L61 residue by generating and testing all possible natural amino acid substitutions at this residue (H3-L61X mutants) to determine its contribution in ensuring proper Spt16−chromatin interactions. As the H3-L61W mutant also causes cryptic intragenic transcription initiation at the *FLO8* gene, we have tested the H3-L61X mutants for this defect and have uncovered a correlation between H3-L61X-mediated defects in repression of intragenic transcription initiation and in impairment of Spt16−gene interactions. Our studies shed new light onto the contribution of the H3-L61 residue to chromatin dynamics, particularly in relation to FACT-mediated processes.

## Materials and Methods

### Yeast strains and growth media

All strains used in this study, listed in Table 1, are *GAL2^+^* derivatives of the S288C strain background (Winston *et al.* 1995). Strains yADP83-yADP88 harbor plasmid pDM9, a centromeric *URA3*-marked plasmid containing the *HHT1* and *HHF1* genes described in a previous study (Duina and Winston 2004). Details for the generation of the H3-L61X mutants are provided below. Standard genetic techniques and details for media preparation have been presented previously (Rose *et al.* 1990). Drug-containing plates were prepared using the following concentrations: formamide, 3%; hydroxyurea, 150 mM; and caffeine, 15 mM.

**Table 1:** *Saccharomyces cerevisiae* strains

Strain	Genotype
yADP83	*MAT*a *his3∆200 leu2∆1 ura3**^a^* *lys2-128δ (hht1-hhf1)∆*::*LEU2 KanMX4-GAL1pr-FLO8-HIS3 HHT2* <*pDM9* (H3-WT/H4-WT)*>*
yADP84	*MAT*a *his3∆200 leu2∆1 ura3**^a^* *lys2-128δ (hht1-hhf1)∆*::*LEU2 KanMX4-GAL1pr-FLO8-HIS3 hht2∆*::*TRP1* <*pDM9* (H3-WT/H4-WT)*>*
yADP85	*MAT*a *his3∆200 leu2∆1 ura3**^a^* *lys2-128δ (hht1-hhf1)∆*::*LEU2 KanMX4-GAL1pr-FLO8-HIS3 hht2*(H3-L61P) <*pDM9* (H3-WT/H4-WT)*>*
yADP86	*MAT*a *his3∆200 leu2∆1 ura3**^a^* *lys2-128δ (hht1-hhf1)∆*::*LEU2 KanMX4-GAL1pr-FLO8-HIS3 hht2*(H3-L61R) <*pDM9* (H3-WT/H4-WT)*>*
yADP87	*MAT*a *his3∆200 leu2∆1 ura3**^a^* *lys2-128δ (hht1-hhf1)∆*::*LEU2 KanMX4-GAL1pr-FLO8-HIS3 hht2*(H3-L61D) <*pDM9* (H3-WT/H4-WT)*>*
yADP88	*MAT*a *his3∆200 leu2∆1 ura3**^a^* *lys2-128δ (hht1-hhf1)∆*::*LEU2 KanMX4-GAL1pr-FLO8-HIS3 hht2*(H3-L61E) <*pDM9* (H3-WT/H4-WT)*>*
yADP89	*MAT*a *his3∆200 leu2∆1 ura3**^a^* *lys2-128δ (hht1-hhf1)∆*::*LEU2 KanMX4-GAL1pr-FLO8-HIS3 HHT2*
yADP90	*MAT*a *his3∆200 leu2∆1 ura3**^a^* *lys2-128δ (hht1-hhf1)∆*::*LEU2 KanMX4-GAL1pr-FLO8-HIS3 hht2*(H3-L61M)
yADP91	*MAT*a *his3∆200 leu2∆1 ura3**^a^* *lys2-128δ (hht1-hhf1)∆*::*LEU2 KanMX4-GAL1pr-FLO8-HIS3 hht2*(H3-L61F)
yADP92	*MAT*a *his3∆200 leu2∆1 ura3**^a^* *lys2-128δ (hht1-hhf1)∆*::*LEU2 KanMX4-GAL1pr-FLO8-HIS3 hht2*(H3-L61C)
yADP93	*MAT*a *his3∆200 leu2∆1 ura3**^a^* *lys2-128δ (hht1-hhf1)∆*::*LEU2 KanMX4-GAL1pr-FLO8-HIS3 hht2*(H3-L61G)
yADP94	*MAT*a *his3∆200 leu2∆1 ura3**^a^* *lys2-128δ (hht1-hhf1)∆*::*LEU2 KanMX4-GAL1pr-FLO8-HIS3 hht2*(H3-L61Q)
yADP95	*MAT*a *his3∆200 leu2∆1 ura3**^a^* *lys2-128δ (hht1-hhf1)∆*::*LEU2 KanMX4-GAL1pr-FLO8-HIS3 hht2*(H3-L61N)
yADP96	*MAT*a *his3∆200 leu2∆1 ura3**^a^* *lys2-128δ (hht1-hhf1)∆*::*LEU2 KanMX4-GAL1pr-FLO8-HIS3 hht2*(H3-L61S)
yADP97	*MAT*a *his3∆200 leu2∆1 ura3**^a^* *lys2-128δ (hht1-hhf1)∆*::*LEU2 KanMX4-GAL1pr-FLO8-HIS3 hht2*(H3-L61A)
yADP98	*MAT*a *his3∆200 leu2∆1 ura3**^a^* *lys2-128δ (hht1-hhf1)∆*::*LEU2 KanMX4-GAL1pr-FLO8-HIS3 hht2*(H3-L61I)
yADP99	*MAT*a *his3∆200 leu2∆1 ura3**^a^* *lys2-128δ (hht1-hhf1)∆*::*LEU2 KanMX4-GAL1pr-FLO8-HIS3 hht2*(H3-L61Y)
yADP100	*MAT*a *his3∆200 leu2∆1 ura3**^a^* *lys2-128δ (hht1-hhf1)∆*::*LEU2 KanMX4-GAL1pr-FLO8-HIS3 hht2*(H3-L61E)
yADP101	*MAT*a *his3∆200 leu2∆1 ura3**^a^* *lys2-128δ (hht1-hhf1)∆*::*LEU2 KanMX4-GAL1pr-FLO8-HIS3 hht2*(H3-L61H)
yADP102	*MAT*a *his3∆200 leu2∆1 ura3**^a^* *lys2-128δ (hht1-hhf1)∆*::*LEU2 KanMX4-GAL1pr-FLO8-HIS3 hht2*(H3-L61V)
yADP103	*MAT*a *his3∆200 leu2∆1 ura3**^a^* *lys2-128δ (hht1-hhf1)∆*::*LEU2 KanMX4-GAL1pr-FLO8-HIS3 hht2*(H3-L61T)
yADP104	*MAT*a *his3∆200 leu2∆1 ura3**^a^* *lys2-128δ (hht1-hhf1)∆*::*LEU2 KanMX4-GAL1pr-FLO8-HIS3 hht2*(H3-L61K)
yADP105	*MAT*a *his3∆200 leu2∆1 ura3**^a^* *lys2-128δ (hht1-hhf1)∆*::*LEU2 KanMX4-GAL1pr-FLO8-HIS3 hht2*(H3-L61W)
yADP106	*MAT*a *his3∆200 leu2∆1 ura3-52 trp1∆63 lys2-128δ hht2∆*::*URA3-TRP1*
yADP107	*MAT*α *his3∆200 leu2∆1 ura3**^a^* *lys2-128δ (hht1-hhf1)∆*::*LEU2 KanMX4-GAL1pr-FLO8-HIS3 HHT2*

a The allele at this locus is either *ura3-52* or *ura3∆0*.

### Generation of strains expressing H3-L61X mutants

**Figure 1:**
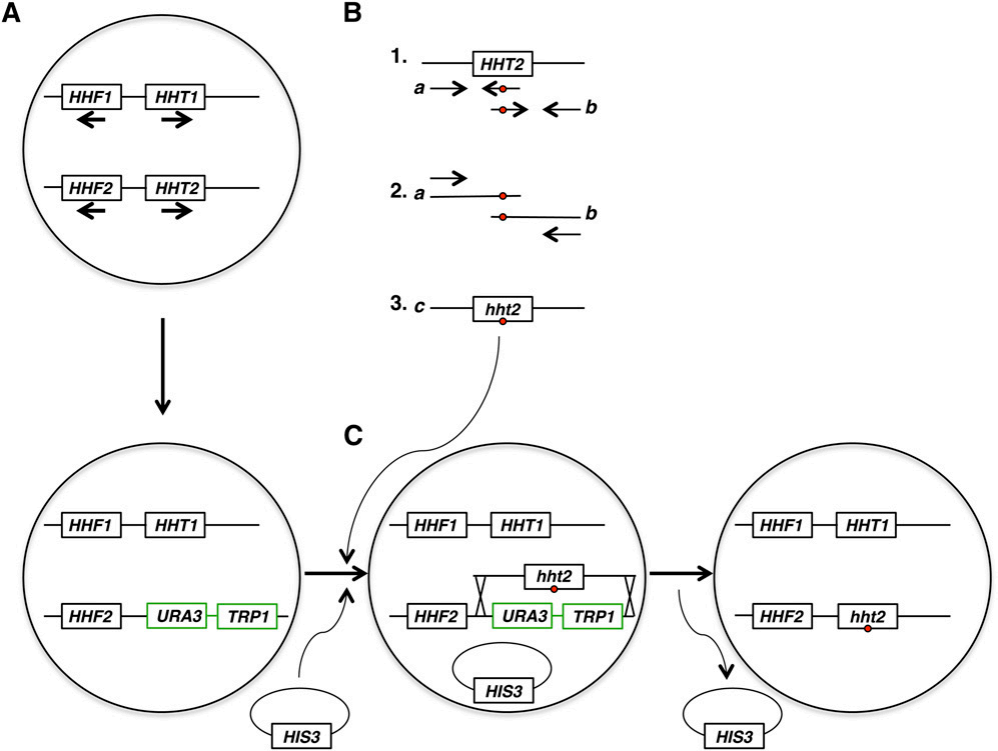
Strategy for the generation of yeast strains expressing histone H3-L61X mutant proteins from the endogenous *HHT2* locus. (A) Top: the haploid yeast genome harbors two sets of divergently transcribed genes encoding histones H3 and H4 (histone H4 is encoded by *HHF1* and *HHF2* and histone H3 is encoded by *HHT1* and *HHT2*—the open reading frames are represented by rectangles and the direction of transcription for each gene is indicated by the corresponding arrow). Bottom: the *HHT2* gene was replaced with the nutritional markers *URA3* and *TRP1* through homologous recombination to generate strain yADP106. (B) Two overlapping segments of the wild-type *HHT2* gene are amplified by PCR as illustrated in diagram 1. The reverse primer (left-pointing arrow) used to generate PCR product ***a*** includes substitution(s) of one, two, or three nucleotides (red circle) within the *HHT2* codon encoding L61 to alter its code to specify for one of the other nineteen amino acids. The forward primer (right-pointing arrow) used to generate PCR product ***b*** includes the analogous substitution(s) (but in the sense orientation, also indicated by a red circle) as that/those present in the reverse primer used for the generation of product ***a***. PCR products ***a*** and ***b*** are then used as templates for the PCR reaction shown in diagram 2. Template-switching events during the PCR can produce DNA molecules that span both fragments ***a*** and ***b***, which can then serve as templates for the generation of full length PCR product ***c*** as shown in diagram 3. (C) Strain yADP106 is co-transformed with PCR products ***c*** and with a plasmid harboring the *HIS3* nutritional marker. Cells from the transformation reactions are plated on media lacking histidine, and resulting colonies are then screened for 5-FOA resistance and for a Ura^-^/Trp^-^ phenotype to identify cells that have replaced the *URA3* and *TRP1* markers with PCR product ***c*** through homologous recombination—the plasmid is subsequently lost through mitotic cell division. We find that selecting for the most “transformable” cells based on their His^+^ phenotype combined with screening for 5-FOA resistance and loss of both *URA3* and *TRP1* markers greatly increases the success rate for obtaining the desired recombination products compared to direct selection on 5-FOA medium. The resulting *hht2* mutant cells are then crossed with cells deleted for *HHT1* to give rise to haploid cells harboring the *hht2* mutant gene as sole source of histone H3. See the *Materials and Methods* section for more details on this strategy. PCR, polymerase chain reaction.

Polymerase chain reaction (PCR) products spanning the entire *HHT2* gene and harboring specific mutations at the H3-L61−encoding codon were generated for each of the 19 H3-L61X−encoding genes using the strategy shown in Figure 1. Generation of ***a*** fragments (see Figure 1B) was accomplished using forward primer OAD20 and the appropriate reverse primer L61Xrev (where X is specific for each of the desired mutations—refer to Table 2 for a list of all primers used for these experiments). Generation of ***b*** fragments was accomplished using the corresponding forward primer L61Xfor and reverse primer OAD21. The template for these reactions was genomic DNA originating from an *HHT2*-wild-type strain. Amplification reactions were carried out using PrimeSTAR HS DNA Polymerase (Takara, Code No. R010A). In each case, the ***c*** fragments were obtained as a result of template-skipping events during PCR amplification reactions that included primers OAD20 and OAD21 (or primers OAD479 and OAD480), the appropriate set of fragments ***a*** and ***b*** (dissolved in melted low-melting point agarose) as templates, and PrimeSTAR HS DNA Polymerase.

**Table 2:** Oligonucleotide Primers

Name	Sequence (5′ to 3′)*^a^*
OAD20	GCGTTCATTATCGCCCAATGTG
OAD21	GCGCTTGATCAGCAGTTCATCG
OAD476	GAAACTATTGGCACGCCCTA
OAD477	CCTGCGAATCAACCGATACT
OAD479	TATGGCTCGGTGTCAAAACA
OAD480	CATGGTTTCTTGCCGGTTAT
L61Mfor	CCAAAAATCTACTGAACTGaTGATCAGAAAGTTACC
L61Mrev	GGTAACTTTCTGATCAtCAGTTCAGTAGATTTTTGG
L61Ffor	CCAAAAATCTACTGAACTGTTcATCAGAAAGTTACC
L61Frev	GGTAACTTTCTGATgAACAGTTCAGTAGATTTTTGG
L61Cfor	CCAAAAATCTACTGAACTGTgtATCAGAAAGTTACC
L61Crev	GGTAACTTTCTGATacACAGTTCAGTAGATTTTTGG
L61Gfor	CCAAAAATCTACTGAACTGggGATCAGAAAGTTACC
L61Grev	GGTAACTTTCTGATCccCAGTTCAGTAGATTTTTGG
L61Qfor	CCAAAAATCTACTGAACTGcaGATCAGAAAGTTACC
L61Qrev	GGTAACTTTCTGATCtgCAGTTCAGTAGATTTTTGG
L61Nfor	CCAAAAATCTACTGAACTGaatATCAGAAAGTTACC
L61Nrev	GGTAACTTTCTGATattCAGTTCAGTAGATTTTTGG
L61Sfor	CCAAAAATCTACTGAACTGTcGATCAGAAAGTTACC
L61Srev	GGTAACTTTCTGATCgACAGTTCAGTAGATTTTTGG
L61Afor	CCAAAAATCTACTGAACTGgcGATCAGAAAGTTACC
L61Arev	GGTAACTTTCTGATCgcCAGTTCAGTAGATTTTTGG
L61Ifor	CCAAAAATCTACTGAACTGaTtATCAGAAAGTTACC
L61Irev	GGTAACTTTCTGATaAtCAGTTCAGTAGATTTTTGG
L61Yfor	CCAAAAATCTACTGAACTGTatATCAGAAAGTTACC
L61Yrev	GGTAACTTTCTGATatACAGTTCAGTAGATTTTTGG
L61Efor	CCAAAAATCTACTGAACTGgaGATCAGAAAGTTACC
L61Erev	GGTAACTTTCTGATCtcCAGTTCAGTAGATTTTTGG
L61Hfor	CCAAAAATCTACTGAACTGcatATCAGAAAGTTACC
L61Hrev	GGTAACTTTCTGATatgCAGTTCAGTAGATTTTTGG
L61Tfor	CCAAAAATCTACTGAACTGacGATCAGAAAGTTACC
L61Trev	GGTAACTTTCTGATCgtCAGTTCAGTAGATTTTTGG
L61Kfor	CCAAAAATCTACTGAACTGaaGATCAGAAAGTTACC
L61Krev	GGTAACTTTCTGATCttCAGTTCAGTAGATTTTTGG
L61Wfor	CCAAAAATCTACTGAACTGTgGATCAGAAAGTTACC
L61Wrev	GGTAACTTTCTGATCcACAGTTCAGTAGATTTTTGG
L61Pfor	CCAAAAATCTACTGAACTGccGATCAGAAAGTTACC
L61Prev	GGTAACTTTCTGATCggCAGTTCAGTAGATTTTTGG
L61Rfor	CCAAAAATCTACTGAACTGcgGATCAGAAAGTTACC
L61Rrev	GGTAACTTTCTGATCcgCAGTTCAGTAGATTTTTGG
L61Dfor	CCAAAAATCTACTGAACTGgatATCAGAAAGTTACC
L61Drev	GGTAACTTTCTGATatcCAGTTCAGTAGATTTTTGG

a The lowercase letters represent nucleotides that differ from those present in the wild-type *HHT2* gene that alter the wild-type H3-L61-encoding codon (TTG) to a different one encoding the corresponding H3-L61X mutant.

The ***c*** fragments were then co-transformed along with the *HIS3*-marked plasmid pRS413 (Sikorski and Hieter 1989) into strain yADP106, which contains the *URA3* and *TRP1* genes in place of the *HHT2* gene (see Figure 1A). His^+^ transformants were then screened for 5-fluoroorotic acid (5-FOA) resistance and inability to grow on media lacking uracil and tryptophan, since these phenotypes indicate successful homologous recombination between the ends of the ***c*** fragments and regions flanking the *URA3-TRP1* genes—the pRS413 plasmid was then lost through mitotic cell division (see Figure 1C). Integration candidates were then analyzed by sequencing both strands of fragments obtained from PCR reactions using the candidates’ genomic DNA as template and primers OAD476 and OAD477, which amplify a region that includes the entire integrated fragment as well as flanking sequences. For all H3-L61X strains used for subsequent experiments, sequencing of the corresponding OAD476-OAD477−amplified fragments revealed the expected mutations at the H3-L61X codon and no additional mutations. Strains yADP90-yADP105 were obtained through genetic crosses between the H3-L61X strains described above and yADP107. Strains yADP83-yADP88 were obtained through similar genetic crosses but with a version of yADP107 that harbors the pDM9 plasmid.

### Chromatin immunoprecipitation (ChIP) assays

ChIP assays and real-time quantitative (q)PCR experiments to determine Spt16 occupancy at the different regions tested in this study were conducted using rabbit polyclonal antibodies specific for Spt16 (a gift from Tim Formosa) as previously described (Myers *et al.* 2011). The primers used for amplification of the specific genomic regions assayed by real-time qPCR were as follows: *PMA1* 5′ region, OAD394 and OAD395; *PMA1* internal region, OAD416 and OAD417; *PMA1* 3′ region, OAD383 and OAD384; *FBA1* 5′ region, OAD419 and OAD420; *FBA1* internal region, OAD421 and OAD422; *FBA1* 3′ region, OAD423 and OAD424; and nontranscribed region on chromosome V (also referred to as NO ORF region), OAD377 and OAD378. The sequences for these primers have been described in previous studies (Myers *et al.* 2011; Nguyen *et al.* 2013).

### Visualization of the nucleosomal region of interest and modeling of H3-L61X mutants

The yeast nucleosome structures shown in Figure 6 were obtained using the PyMOL Molecular Graphics System, Version 1.5.0.3 Schrödinger, LLC using structural information obtained in a previous study (White *et al.* 2001) and available at the Research Collaboratory for Structural Bioinformatics protein data bank (PDB ID:1id3). In each case, modeling of an amino acid substitution at position 61 of histone H3 was accomplished using the Mutagenesis function in PyMOL and using the rotamer with the highest percentage value.

## Results

### Generation and initial functional evaluation of H3-L61X mutants

**Figure 2:**
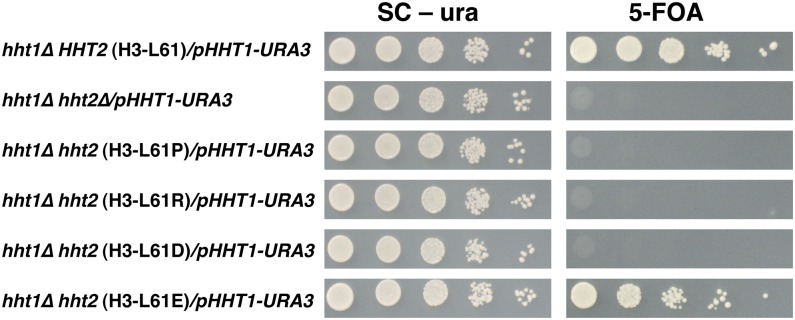
The H3-L61P, H3-L61R, and H3-L61D mutants are unable to support viability when expressed as sole source of histone H3 in cells. Cells of the indicated genotypes and carrying a *URA3*-marked plasmid carrying a wild-type copy of *HHT1* (*pHHT1-URA3*) were grown on nonselective medium for several generations to allow for loss of the plasmid. Following collection, cells were spotted onto SC-ura and 5-FOA plates in a 10-fold dilution series, in which the most concentrated spot (left-most spot on each panel) consisted of ∼240,000 cells. The plates were then placed at 30° and photographed after approximately 2 d (SC-ura) or 3 d (5-FOA) of incubation. Absence of growth on the 5-FOA plate reflects an inability of cells to grow in the absence of the plasmid, thus indicating that the corresponding *hht2* allele cannot support viability when present as sole source of histone H3. Note that cells expressing the H3-L61P, H3-L61R, or H3-L61D mutant display a 5-FOA−sensitive phenotype indistinguishable from that seen in *hht2∆* cells, whereas cells expressing wild-type histone H3 or the H3-L61E mutant (which confers viability as sole source of histone H3) show robust growth on 5-FOA medium. The strains used in these experiments were yADP83-yADP88. 5-FOA, 5-fluoroorotic acid.

To investigate the effects of amino acid substitutions at position 61 of histone H3 on various cellular processes, we first generated haploid yeast cells carrying targeted mutations at the endogenous *HHT2* locus (one of the two histone H3-encoding genes in the haploid yeast genome) using the strategy shown in Figure 1. These experiments gave rise to 19 yeast strains, each expressing a version of histone H3 with a different substituted amino acid at position 61 (referred herein as H3-L61X mutants), collectively representing all possible natural amino acid substitutions at this position. The presence of a wild-type copy of *HHT1*, the other gene encoding histone H3 in yeast, ensured that any strain expressing an H3-L61X mutant unable to support viability on its own could nevertheless be generated and analyzed. Each of the 19 mutant strains was then crossed with a strain deleted for *HHT1* to yield spores whose sole source of histone H3 was derived from the H3-L61X encoding gene. Results from these experiments showed that 16 of the 19 H3-L61X mutants are able to support life when present as only source of histone H3 in cells (see Figure 4), and provided initial evidence that three mutants—H3-L61P, H3-L61R, and H3-L61D—are unable to do so, as viable *hht1∆ hht2* double mutants were not recovered from their respective crosses. Subsequent viability tests confirmed that these three mutants are indeed unable to support life in the absence of a wild-type source of histone H3 (Figure 2).

### Effects of H3-L61X mutants on Spt16−gene interactions

Previous studies have shown that the H3-L61W mutant perturbs association of the transcription elongation factor Spt16 across transcribed genes, causing a marked shift in its distribution toward the 3′ end of transcribed genes (Duina *et al.* 2007; Nguyen *et al.* 2013). To investigate the effects of the newly generated H3-L61X mutants on Spt16−gene interactions, we performed ChIP assays to monitor the binding pattern of Spt16 across two constitutively transcribed genes in the context of the H3-L61X mutants [note that these and all subsequent experiments have been carried out using haploid strains deleted for *HHT1* and for *HHF1* (one of two genes encoding histone H4 in yeast); therefore, these strains express histone H3 and H4 each from a single gene, with histone H3 being either wild-type or carrying one of the substitutions at position 61 able to confer viability —refer to Table 1].

**Figure 3:**
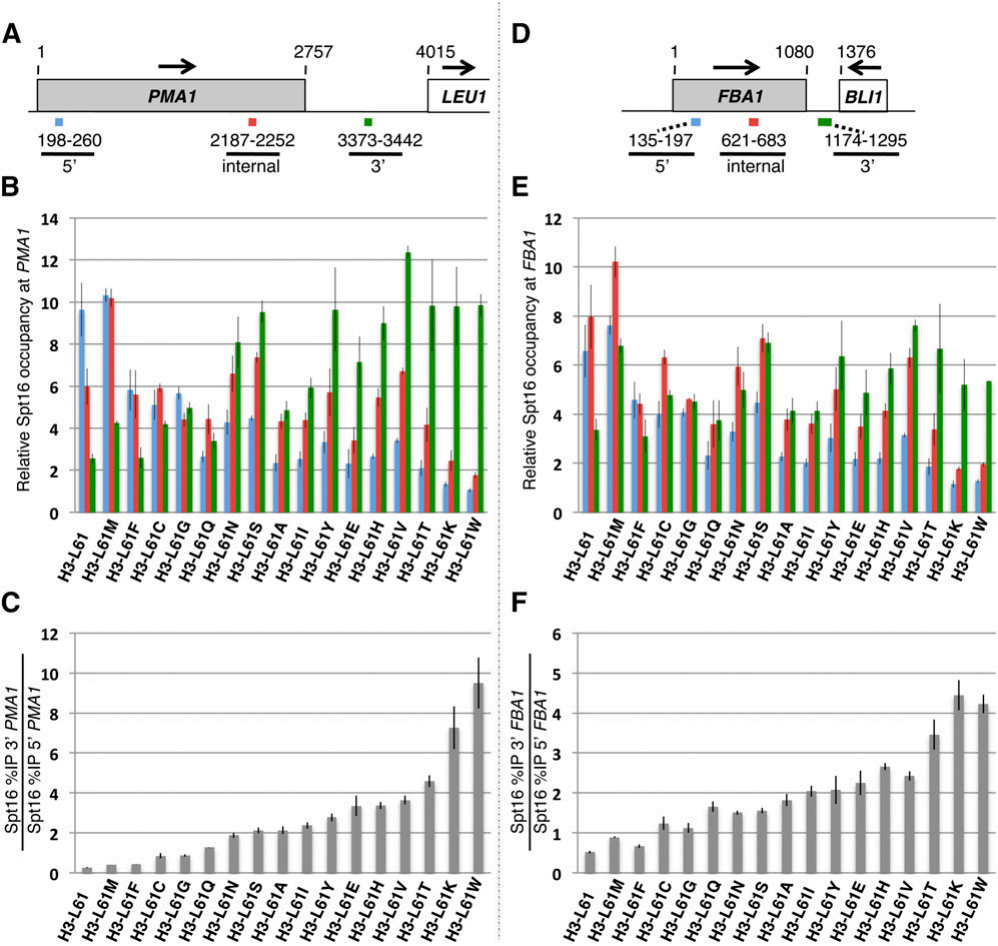
Effects of H3-L61X mutants on Spt16 occupancy across the *PMA1* and *FBA1* genes. (A,D). Schematic representations of genomic regions encompassing the *PMA1* and *FBA1* genes adapted from diagrams used in a previous study (Nguyen *et al.* 2013). The arrows indicate the direction of transcription of the corresponding genes and the numbers reflect nucleotide positions, with 1 corresponding to the “A” nucleotide at the beginning of the corresponding *PMA1* or *FBA1* open reading frames. The 5′, internal, and 3′ locations correspond to the regions assayed in real-time qPCR experiments following the chromatin immunoprecipitation (ChIP) procedure (see *Materials and Methods*). (B,E) Results from Spt16 ChIP assays expressed as a ratio of the %IP of the indicated *PMA1* or *FBA1* regions to the %IP of a non-transcribed region on chromosome V (see *Materials and Methods*). (C,F) Results from the same ChIP experiments that gave rise to the data shown in panels B and E, but expressed as the ratio of the %IP of the 3′ region to the %IP of the 5′ region at *PMA1* or *FBA1*. Representation of the ChIP data in this fashion facilitates the determination of the overall 5′ to 3′ shift in the distribution of Spt16 across the gene of interest. For the data shown in panels C and F, statistically significant differences in Spt16 distribution were established in all cases (Student’s *t*-tests; *P* < 0.05), except for the H3-L61F mutant at *FBA1* (*P* = 0.056). For all charts in this figure, the H3-L61X mutants tested are shown on the X-axes and are arranged in increasing order of strength of mutant phenotype based on the values for the bar graphs shown in panel C. The data are expressed as the mean ± SEM from at least three independent experiments. The strains used in these experiments were yADP89-yADP105.

As shown in Figure 3, all 16 viable H3-L61X mutants cause statistically significant defects in Spt16 interactions at *PMA1* and, with the exception of the H3-L61F mutant, at *FBA1* as well. Interestingly, the mutant that confers the strongest defects in these assays is H3-L61W, which is the mutant that was identified and tested in our original studies (Duina and Winston 2004; Duina *et al.* 2007). As previously shown for H3-L61W (Duina *et al.* 2007; Nguyen *et al.* 2013), the H3-L61X mutants cause an overall shift in Spt16 occupancy toward the 3′ ends of the *PMA1* and *FBA1* genes (see Figure 3, C and F). These results underscore the importance of the H3-L61 residue in contributing to proper Spt16−gene interactions. The defects conferred by the H3-L61X mutants on Spt16−gene interactions range from very mild to severe, and their relative degree of severity is comparable at the *PMA1* and the *FBA1* genes (compare Figure 3, C and F), suggesting that the mechanism that underlies Spt16−histone H3 interactions is similar at these genes and that specific H3-L61X mutants interfere with this mechanism similarly at both genes. Since previous work has shown that H3-L61W, the most defective mutant, causes relatively minor perturbations in nucleosome occupancy at the *PMA1-LEU1* region (Duina *et al.* 2007), we consider it unlikely that the H3-L61X mutants mediate their effects on Spt16−gene interactions through alteration in overall abundance of nucleosomes across genes.

An analysis of the results from these experiments offers insights into the features of the H3-L61 residue important for promoting proper Spt16−gene interactions. First, its hydrophobic nature appears to be critical as hydrophilic amino acid substitutions at position 61 are among the ones causing the strongest defects. This finding is consistent with the observation that whereas the H3-L61F mutant causes minor defects in Spt16 interactions with *PMA1* and *FBA1*, H3-L61Y causes much more pronounced perturbations, possibly due to the hydrophilic nature of the hydroxyl group on tyrosine. Second, the finding that substitutions at position 61 with either isoleucine or valine—both of which might be expected to be well tolerated in place of leucine—cause strong defects in these assays could indicate that the precise structural conformation adopted by H3-L61 is required for proper Spt16−gene interactions and that isoleucine and valine—both C-beta branched amino acids—are unable to adopt such conformation due their more limited rotational freedom across the polypeptide backbone. Third, the size of the amino acid side chain at position 61 also seems to be relevant as a substitution with the hydrophobic but much bulkier amino acid tryptophan causes the strongest perturbations in Spt16−gene interactions. Attributes shared between leucine and the substitutions that confer the smallest defects—methionine, phenylalanine, and cysteine—are hydrophobicity, normal flexibility, and lack of excessive bulk.

### Analysis of growth phenotypes of H3-L61X mutants

**Figure 4:**
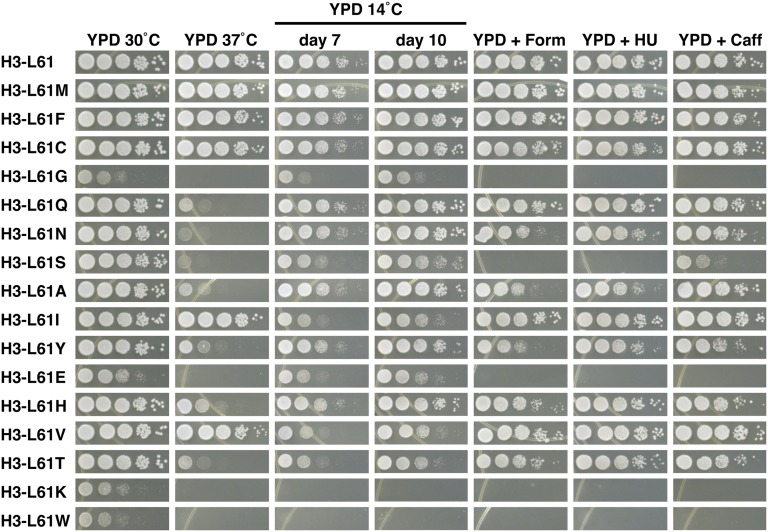
Phenotypes of cells expressing H3-L61X mutants subjected to a variety of growth conditions. Cells expressing the indicated histone H3 proteins as sole source of histone H3 were grown overnight on YPD medium and subsequently collected and spotted on the indicated plates (for each condition tested, all mutants were spotted on the same plate) in a 10-fold dilution series, with the most concentrated spots (left-most spot in each panel) consisting of ∼120,000 cells. Plates were placed at the indicated temperatures (note that the drug-containing plates were incubated at 30°), and photographed after the following approximate incubation times: YPD 30°, 2 d; YPD 37°, 2 d; YPD 14°, 7 and 10 d, as indicated; YPD + formamide (Form), 3 d; YPD + hydroxyurea (HU), 3 d; YPD + caffeine (Caff), 5 d. The YPD 14° plate is shown at two different incubation times to allow for assessment of subtle cold-sensitive phenotypes (*e.g.*, for the H3-L61H mutant). Mutants are presented in the same order as that shown in Figure 3. The growth patterns shown in this figure were closely reproduced in an independent experiment for all samples except for H3-L61G, which showed modestly stronger growth on the YPD 30° plate. Additional growth experiments showed that the variable growth phenotype conferred by H3-L61G is an inherent property of this mutant. The strains used in these experiments were yADP89-yADP105. YPD, yeast extract peptone dextrose.

Previous analyses have shown that the H3-L61W mutant displays several abnormal growth phenotypes (Duina and Winston 2004; Duina *et al.* 2007). To more fully characterize the newly generated histone H3 mutants and to assess possible correlations between growth defects and the defects in Spt16−gene interactions as determined by the ChIP experiments described in the previous section, we tested the ability of the viable H3-L61X mutants to grow under a variety of conditions using serial dilution spot test assays (Figure 4).

Results from these experiments show that most of the mutants display growth defects at high and low temperatures, as well as other mutant phenotypes. With the exception of the H3-L61I and H3-L61V mutants, there appears to be a general correlation between impaired growth at 37° and perturbations in Spt16−gene interactions (Figure 4: compare growth at 37° of the weakest Spt16−gene interaction mutants H3-L61M, -L61F, and -L61C with the other H3-L61X mutants). The observation that H3-L61I and H3-L61V mutants do not show a temperature sensitive phenotype at 37° might indicate that although the structural rigidity imparted by isoleucine and valine at position 61 of histone H3 interferes with Spt16−interactions, it does not cause cells to become sensitive to elevated temperatures. Conversely, disruptions of Spt16−gene interactions through introduction of polarity/charge or bulk at position 61 also cause cells to display a temperature sensitive phenotype at 37°. A general correlation between cold sensitivity and defects in Spt16−gene interactions can also be seen, as H3-L61X mutants that cause moderate to strong perturbations in Spt16−gene interactions show a range of sensitivities to the low temperature of 14° (Figure 4).

Sensitivities of H3-L61X mutants to the drugs formamide, hydroxyurea, and caffeine range from none to very strong and they do not show a clear correlation with impairment in Spt16−gene interactions (Figure 3 and Figure 4). In most cases, H3-L61X mutants that show sensitivity to one drug also show sensitivity to the other two drugs suggesting that these sensitivities share a common underlying mechanism.

### Assessment of effects of H3-L61X mutants on phenotypes indicative of transcription and chromatin defects

**Figure 5:**
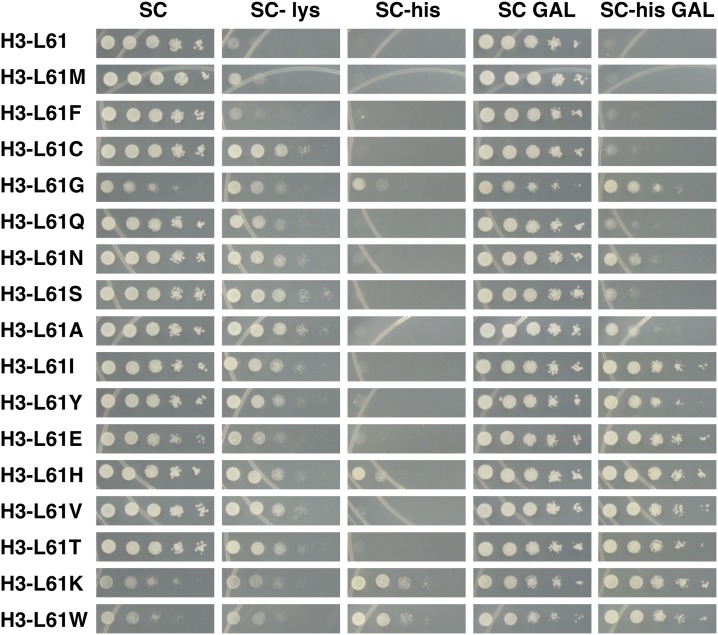
Effects of H3-L61X mutants on growth phenotypes indicative of transcription and chromatin defects. Cells expressing the indicated H3-L61X mutants were treated as described in Figure 4, except that the most concentrated spots consisted of ∼240,000 cells. Plates were placed at 30°C and photographed after the following approximate times of incubation: SC, 2 d; SC-lys, 2 d; SC-his, 3 d; SC GAL, 3 d; SC-his GAL, 3 d. The growth patterns shown in this figure were closely reproduced in an independent experiment for all samples except for H3-L61G, which, as was the case in the experiments described in Figure 4, showed some variability: this mutant showed modestly stronger growth on the SC and SC GAL plates and weaker growth on the SC-his plate. The strains used in these experiments were yADP89-yADP105. SC, SC-lys, and SC-his plates contained glucose and SC GAL and SC-his GAL plates contained galactose as carbon and energy source.

We continued the characterization of the newly generated H3-L61X mutants by testing them for phenotypes associated with defects in transcription and chromatin function. In one set of experiments, H3-L61X mutant cells carrying the *lys2-128δ* allele were assayed for growth on medium lacking lysine as the ability to grow under these conditions—a phenotype referred to as Spt^−^—is indicative of perturbations in transcription and chromatin structure (Winston 1992). As shown in Figure 5, with the exception of H3-L61F, all other H3-L61X mutants display a range of Spt^−^ phenotypes, thus underscoring the importance of L61 of histone H3 in maintaining chromatin integrity. The strength of the Spt^−^ phenotypes conferred by the H3-L61X mutants does not correlate with the strength of the defects they impart on Spt16−gene interactions (for example, compare the Spt^−^ phenotypes of the H3-L61C and H3-L61T mutants in Figure 5 with the perturbations they impart on Spt16−gene interaction in Figure 3), suggesting that the two phenotypes are not mechanistically related to each other.

In a second set of experiments we made use of the *FLO8-HIS3* reporter gene, which contains the *HIS3* coding region fused downstream from a cryptic transcription initiation site within the *FLO8* coding region (Cheung *et al.* 2008), to monitor for intragenic transcription initiation events. Since *FLO8-HIS3* is under the control of the inducible *GAL1* promoter, intragenic transcription initiation events can be easily monitored by assaying cells—otherwise deleted for the endogenous *HIS3* gene—for growth on media lacking histidine in the context of high transcription levels (*i.e.*, in the presence of galactose) or in the context of little to no transcription (*i.e.*, in the presence of glucose). As previously reported (Myers *et al.* 2011; Nguyen *et al.* 2013), the H3-L61W mutant confers a strong His^+^ phenotype on media containing either glucose or galactose (Figure 5). Although a few H3-L61X mutant strains behave indistinguishably from wild-type cells on media lacking histidine, most show His^+^ phenotypes ranging from mild to strong (Figure 5). Members of this latter class of mutants display stronger growth on galactose-containing medium (SC-his GAL) compared to glucose-containing medium (SC-his), suggesting that the chromatin defects leading to intragenic transcription initiation imparted by these H3-L61X mutants are more pronounced in the context of high levels of transcription (Figure 5). Interestingly, we see a general correlation between H3-L61X mutants’ ability to confer growth on SC-his GAL medium and the degree of the defects they impart on Spt16−gene interactions as assessed by the ChIP experiments shown in Figure 3. For example, the eight mutants that confer the strongest defects in Spt16−gene interactions (H3-L61I, Y, E, H, V, T, K, and W) are also the ones that show the most robust growth on SC-his GAL plates. Taken together, these results suggest that the defects in repression of intragenic transcription initiation and in Spt16−gene interactions caused by mutations at position 61 of histone H3 are in some manner mechanistically linked to each other.

## Discussion

In this work, we have carried out a systematic mutational analysis of the H3-L61 residue to gain insights into its contribution to chromatin function. Our finding that substitutions of H3-L61 to each of the remaining 19 natural amino acids confer defects in one or more of the phenotypes we have assayed highlights the importance of H3-L61 in maintaining chromatin integrity and is consistent with the high degree of evolutionary conservation of this residue across eukaryotic organisms (we note that this level of conservation is not unique to the L61 residue as most other histone H3 residues are also highly conserved). An evaluation of the nature of the defects conferred by each of the H3-L61X mutants indicates that features of H3-L61 important for maintaining proper nucleosome functions include its hydrophobicity, its flexibility across the polypeptide backbone, and its lack of excessive bulk. The aliphatic nature of H3-L61 does not appear to be critical for these functions as a mutation to the aromatic amino acid phenylalanine did not cause strong defects in our assays. We also provide evidence that H3-L61X−dependent defects in Spt16−gene interaction and in repression of intragenic transcription initiation are mechanistically linked to each other.

**Figure 6:**
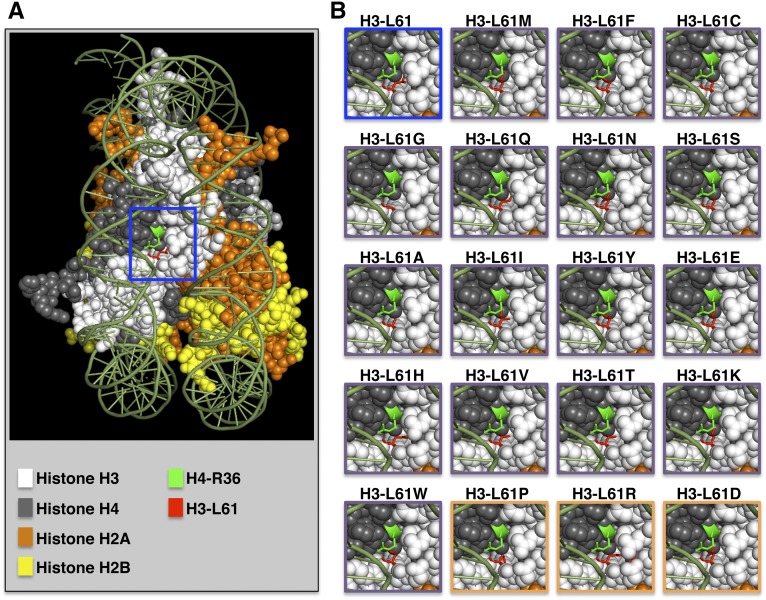
Location of the H3-L61 and H4-R36 residues in the nucleosome and modeling of the H3-L61X mutants. (A) Side-view of the yeast nucleosome core particle with the DNA shown in olive green and the histones color-coded as indicated in the figure. The H3-L61 and H4-R36 residues are shown in stick representation and color-coded as indicated. The blue box represents the region analyzed in panel B. (B) Close-up views of the nucleosomal region indicated by the blue box in panel A showing the wild-type structure (top-left image with blue border) and models of the H3-L61X substitutions. The images with purple borders correspond to the H3-L61X mutants able to confer viability when expressed a sole source of histone H3 in cells and are shown in the same order as that used for the experiments shown in Figure 3, Figure 4, and Figure 5. Models of the H3-L61X mutants unable to support viability in the absence of wild-type histone H3 (see Figure 2) are framed by orange borders. The H3-L61X models were generated using PyMOL and show the orientation of the substituted residues based on the rotamers with greatest frequencies found in other proteins as calculated by the software program.

In previous work, we showed that an H4-R36A mutant perturbs Spt16 association across genes in a fashion similar to that seen in the context of H3-L61W, and genetic evidence indicated that these two histone mutants affect Spt16−gene interactions through a common mechanism (Nguyen *et al.* 2013). H4-R36 and H3-L61 are adjacent to each other on the side of the nucleosome, with the former being located on the surface making direct contacts with DNA and the latter being buried into the particle ((Luger *et al.* 1997; Luger and Richmond 1998) and Figure 6A). How do these histone residues contribute to proper Spt16−chromatin interactions, and how might they be functionally linked? Based on our original studies, we considered a model in which H4-R36 is part of a nucleosomal surface that interacts with Spt16, and mutations that perturb this surface can lead to abnormal Spt16 interactions across genes. In the context of this model, we envisioned two possible mechanisms by which H3-L61W could affect H4-R36 function: (i) the π electrons of the aromatic ring of the tryptophan residue could interfere with the ability of H4-R36 to form ionic bonds with other factors, or (ii) the integrity of the hydrophobic microenvironment normally occupied by H3-L61 is required for proper positioning of H4-R36 for interactions with other factors, and the tryptophan residue in H3-L61W disrupts this microenvironment through its bulky nature. The results from the present study do not support the first mechanism, as the associated predictions that substitutions of H3-L61 with the aromatic amino acid phenylalanine should cause strong perturbations in Spt16−gene interactions while substitutions with aliphatic hydrophobic residues (*e.g.*, alanine and isoleucine) should not, were not substantiated. Our results are instead consistent with the second mechanism, as H3-L61X mutants (modeled in Figure 6B) that are predicted to disrupt the H3-L61 microenvironment through the introduction of polarity/charge or bulk or by interfering with H3 polypeptide flexibility all cause strong perturbations in Spt16−gene interactions. The H3-L61X mutants’ effects on Spt16−chromatin interactions are likely to be for the most part specific, as previous work has shown that H3-L61W, the strongest H3-L61X mutant, does not significantly affect Pol II− and Spt4−gene interactions and only modestly affects Spt6−gene interactions (Duina *et al.* 2007; Lloyd *et al.* 2009). The isolation of Spt16 mutants that can suppress H3-L61W−dependent defects in Spt16−chromatin interactions (Duina *et al.* 2007; Myers *et al.* 2011) further points to a specific relationship between Spt16 and the H3-L61 residue.

Most H3-L61X mutants cause decreased levels of Spt16 occupancy at the 5′ ends of *PMA1* and *FBA1* while simultaneously elevating the levels of Spt16 occupancy at the 3′ ends of the same genes. In addition, in this study we have provided evidence that the defects in Spt16−gene interactions imparted by H3-L61X mutants are correlated with defects in repression of intragenic transcription initiation. Currently, the molecular mechanism that underlies this relationship is unknown. One possibility is that the primary defect conferred by the H3-L61X mutants is to directly interfere with the ability of Spt16 to dissociate from the 3′ ends of genes at the end of the transcription process. Since this 3′ trapping may be occurring throughout the genome (we have previously shown that H3-L61W affects interactions between Spt16 and all other transcribed genes assayed—a total of six genes (Duina *et al.* 2007)), it could lead to reduced levels of Spt16 available for recruitment to transcribed genes, resulting in lower Spt16 occupancy levels at 5′ ends of genes as well as in defects in Spt16-mediated transcription-coupled nucleosome reassembly over the bodies of genes, which would ultimately lead to intragenic transcription initiation. Alternatively, it is possible that the H3-L61X mutants directly affect chromatin environments over transcribed genes in ways that cause cryptic intragenic transcription initiation, for example, by interfering with proper transcription-coupled nucleosome reassembly, which in turn could in some manner cause a shift in Spt16 distribution toward the 3′ ends of genes. 

We note, however, that cryptic intragenic transcription initiation *per se* is not sufficient for causing a 3′ shift of Spt16 across genes, since in previous work we have isolated several Spt16 mutants that cause strong intragenic transcription initiation but that do not confer significant defects in Spt16 association across the *PMA1* gene (Myers *et al.* 2011). We previously disfavored the possibility that the H3-L61W−dependent defects in Spt16−gene interactions and in repression of intragenic transcription initiation are functionally related to each other in light of the fact that among several Spt16 mutants we isolated, Spt16-E735G was among the strongest suppressors of the H3-L61W−dependent defect in Spt16 distribution across *PMA1*, but was the weakest suppressor of the intragenic transcription initiation defect at *FLO8-HIS3* (Myers *et al.* 2011). However, Spt16-E735G can cause intragenic transcription initiation at *FLO8-HIS3* in a histone H3 wild-type background (Myers *et al.* 2011), and therefore the apparent lack of suppression of the H3-L61W−dependent defect in repression of intragenic transcription could be attributed to Spt16-E735G’s own ability to promote intragenic transcription initiation.

In conclusion, our systematic mutational analysis has shed new light on the contribution of the H3-L61 residue to proper chromatin function. Future studies will focus on further characterizing the nature of the nucleosomal region involved in controlling Spt16 interactions with transcribed genes. It will also be of interest to determine the functional outcomes of Spt16 trapping at the 3′ ends of genes due to histone mutants, in particular in relation to possible effects on nucleosome composition and on transcription termination. Collectively, these and related studies will contribute to our understanding of the mechanisms that govern interactions between Spt16 and nucleosomes during transcription, and possibly, during other chromatin processes that involve Spt16 function, such as DNA replication and repair.

## Supplementary Material

Corrigendum
