# Supplementary material for: A Systematic Mutational Analysis of a Histone H3 Residue in Budding Yeast Provides Insights into Chromatin Dynamics
Source: G3 (Bethesda). 2015 Feb 23;5(5):741–9. doi: 10.1534/g3.115.017376 (PMC4426362; doi:10.1534/g3.115.017376)
Supplement: Corrigendum [file supp_5_5_741__index.html]

Corrigendum 

# A Systematic Mutational Analysis of a Histone H3 Residue in Budding Yeast Provides Insights into Chromatin Dynamics

## Corrigendum for Johnson *et al.*, G3 5 (5) 741-749

**Files in this Data Supplement:**

- Corrigendum
