# Supplementary material for: A Systematic Mutational Analysis of a Histone H3 Residue in Budding Yeast Provides Insights into Chromatin Dynamics
Source: G3 (Bethesda). 2015 Feb 23;5(5):741–9. doi: 10.1534/g3.115.017376 (PMC4426362; doi:10.1534/g3.115.017376)
Supplement: Corrigendum [file supp_g3.115.017376_Corrigendum_Johnson.pdf]

Corrigendum for Johnson *et al.*, *G3* 5 (5) 741-749.

G3, Vol 5, 741-749, May 2015, Copyright © 2015 Genetics Society of America.

CORRIGENDUM

In the article by P. Johnson, V. Mitchell, K. McClure, M. Kellems, S. Marshall, *et al.* (G3 5: 741-749) entitled "A Systematic Mutational Analysis of a Histone H3 Residue in Budding Yeast Provides Insights into Chromatin Dynamics," two of the oligonucleotide primer sequences in Table 2 on page 745 were listed as

L61Hfor ccaaaaatctactgaactgCATatcagaaagttacc
L61Hrev ggtaactttctgatATGcagttcagtagatttttgg

They have been corrected and are now listed as

L61Hfor CCAAAAATCTACTGAACTGcatATCAGAAAGTTACC
L61Hrev GGTAACTTTCTGATatgCAGTTCAGTAGATTTTTGG
